# To the Editors of the Medical and Physical Journal

**Published:** 1803-01-01

**Authors:** W. Cuff

**Affiliations:** Newington


### *To the Editors of the Medical and Physical Journal.*

Gentlemen,

In your Address to Correspondents, in the 41st Number of your Journal, is the following expression : " Dr. Davis, of Bath, complains of injustice in Mr. Cuff's evidence, as detailed in the Report of the Committee on Dr. Jenner's Petition, No. 13, of the Appendix to the Report." Hence I conclude, that you had received a letter from Dr. Davis, containing this complaint, and stating the particular grounds of it; but that you did not think it worthy to appear in your respectable publication.

Having perused a letter of this kind in the Medical and Chirurgical Review, I found some insinuations respecting Dr. Jenner and myself, requiring an answer.

These insinuations are equally scurrilous and illiberal. They tend to call in question, not only my motives for giving such evidence, but also the utility of the practice recommended by Dr. Jenner.

The evidence I gave was, that I did not conceive Dr. Davis had the knowledge of the disease which was necessary; and that he did not know the proper time for taking matter. This opinion I formed from an interview I had with him at Bath, the latter end of the year 1800.

He acknowledged to me, that he had not so much information on the subject as he wished; and that he should be happy to have a little more conversation with me, as he found I knew more of the cow-pox than himself; having been in the habit of delineating the vaccine pustule in its different stages.

Dr. Davis observes, that " the actions of men are often mysterious, until their motives are known."—His own conduct illustrates the truth of his observation. His letter is calculated, not only to defend himself, but also to impeach the character of Dr. Jenner. Yet, I am convinced it will prove harmless to a man, whose name stands too high to need any support from my humble pen ; to a man, who will be handed down to posterity with grateful remembrance, as one of the greatest benefactors of mankind.

I know of no reason, but the want of information, which could induce Dr. Davis to ask a stranger to call on him at his residence in Bath. On my return to London, in January 1801, I repeated the substance of my conversation with Dr. Davis to Dr. Jenner ; at the same time expressing pressing my apprehension that we should hear of some mistakes committed at Bath.

To vindicate my conduct from the invidious insinuations of Dr. Davis, that my evidence was fabricated for the purpose of rebutting some strong information, "which might have been unfavourable to Dr. Jenner's views,"— It is only necessary to remark, that this conversation was communicated by Dr. Jenner to a gentleman at Bath, who is well known in the practice of vaccine inoculation, in September, 1801, six months before it was reported by me to the Committee.

It is not the first time that Dr. Davis has cast reflections on the practice; reflections which, in his hands, it has probably too well deserved. If Dr. Davis was anxious for fair representations, why was a case which occurred in his own practice, communicated by a gentleman who had never seen it, and who, by his mode of description, is obviously unacquainted with the nature of the cow-pox, unless he was apprehensive that his own word would have but little weight?

That Dr. Davis has been in the habit of propagating unfavourable reports concerning vaccine inoculation, the Bath Journals, and your own, bear ample testimony; I would therefore seriously advise him, on his own account, to be more cautious in future, lest, after again libelling the new practice, he should again be obliged to read a recantation of his errors.

Vaccine Inoculation is now too well established to be shaken by the breath of idle rumour, fretful spleen, or disappointed ambition: hence, any failure which may hereafter occur, will be considered as a reflection on the practitioner, and not on the practice.

